# Population pharmacokinetic model of tranexamic acid in patients who undergo cardiac surgery with cardiopulmonary bypass

**DOI:** 10.1007/s00228-025-03802-0

**Published:** 2025-01-16

**Authors:** Tsuyoshi Nakai, Takahiro Tamura, Yasuhiro Miyagawa, Takayuki Inagaki, Masato Mutsuga, Shigeki Yamada, Kiyofumi Yamada, Kimitoshi Nishiwaki, Hiroyuki Mizoguchi

**Affiliations:** 1 Department of Pharmacotherapeutics and Informatics, Fujita Health University School of Medicine, 1-98 Dengakugakubo, Kutsukake-Cho, Toyoake, Aichi 470-1192 Japan; 2 Department of Neuropsychopharmacology and Hospital Pharmacy, Nagoya University Graduate School of Medicine, 65 Tsurumai-Cho, Showa-Ku, Nagoya, 466-8560 Japan; 3 Department of Anesthesiology, Nagoya University Graduate School of Medicine, 65 Tsurumai-Cho, Showa-Ku, Nagoya, 466-8550 Japan; 4 Division of Pharmaceutical Sciences I, Faculty of Pharmacy, Meijo University, 150 Yagotoyama, Tempaku-Ku, Nagoya, 468-8503 Japan; 5 Department of Cardiac Surgery, Nagoya University Graduate School of Medicine, 65 Tsurumai-Cho, Showa-Ku, Nagoya, 466-8550 Japan; 6 Division of Behavioral Neuropharmacology, International Center for Brain Science, Fujita Health University, 1-98 Dengakugakubo, Kutsukake-Cho, Toyoake, Aichi 470-1192 Japan

**Keywords:** Tranexamic acid, Cardiac surgery, Population pharmacokinetic, Cardiopulmonary bypass

## Abstract

**Purpose:**

Tranexamic acid (TXA) is widely used as an antifibrinolytic drug. However, studies to determine the optimal blood concentration of TXA have produced inconsistent results. During cardiac surgery, cardiopulmonary bypass (CPB) has serious effects on drug distribution, elimination, and plasma concentration. Therefore, we aimed to establish a population pharmacokinetics model of TXA in patients undergoing cardiac surgery with CPB that considers renal function as a covariate, thereby facilitating personalized treatment.

**Methods:**

In total, 453 TXA plasma samples were prospectively collected from 77 patients who underwent cardiac surgery with CPB. Plasma concentrations were determined by ultra-performance liquid chromatography-tandem mass spectrometry. The population pharmacokinetic model of TXA was analyzed using nonlinear mixed-effects modeling.

**Results:**

The two-compartment–based model with combined errors was determined as the best. The final model included the effect of bodyweight and CL_cr_ may be summarized as *V*_1_ (L) = 12.77 × (bodyweight / 61.4)^0.911^, *V*_2_ (L) = 6.857, CL_1_ (L/h) = 3.263 × [CL_cr_ (L/h) / 61.0]^0.752^, CL_2_ (L/h) = 2.859.

**Conclusion:**

Patients who undergo cardiac surgery with CPB may require an adjusted dose of TXA tailored to CPB due to lower CL_1_ and increased *V*_1_. Our TXA population pharmacokinetic model may be useful for developing individualized dosing designs for TXA in patients who undergo cardiac surgery with CPB.

**Supplementary Information:**

The online version contains supplementary material available at 10.1007/s00228-025-03802-0.

## Introduction

Antifibrinolytic agents, including aprotinin and lysine analogs, are used worldwide to reduce bleeding and transfusion in patients who undergo high-risk surgery [1–3]. In particular, tranexamic acid (TXA), a lysine analog, is used to reduce the risk of blood loss, blood transfusion, and reoperation in patients who undergo cardiac surgery [4]. In these patients, a cardiopulmonary bypass (CPB) is often used to provide partial or complete support for severe cardiopulmonary dysfunction during surgery [5, 6]. However, the use of CPB is known to cause abnormal platelet function and increased blood coagulation and fibrinolytic reactions because the blood is diluted by filling fluids and cardioprotective solutions and comes into contact with the CPB pump [7]. In addition, hypothermia induces fibrinolytic system activation in patients who undergo cardiac surgery with CPB [7], requiring the use of higher TXA doses than in those who undergo other surgeries. However, administering high TXA doses appears to increase the risk of postoperative neurological events, including seizures and cerebral infarction, owing to reduced cerebral blood flow [8, 9]. Notably, several studies have reported that seizures associated with TXA are dose-related [10–12].

Although a growing number of studies have proposed the effective and safe blood concentration of TXA in clinical practice [4, 13–15], their results are inconsistent. Furthermore, only a handful of studies have reported the population pharmacokinetics (PK) of TXA in patients undergoing cardiac surgery with CPB [13, 16]. However, the evidence for TXA population PK models for personalized medicine remains insufficient owing to the lack of consistent dosing regimens for TXA worldwide. Additionally, it is necessary to establish a TXA PK model that considers renal function because TXA is primarily excreted in the urine.

Therefore, here, we aimed to develop a population PK model of a TXA dosing scheme in patients who undergo cardiac surgery with CPB, which may prove useful for personalized TXA medicine.

## Methods

### Study design and sample preparation

This prospective observational study enrolled patients who received TXA at the Nagoya University Hospital between August 2021 and August 2022. Patients who underwent cardiac surgery with CPB and received intravenous (IV) TXA were selected. We excluded patients who were undergoing hemodialysis, those without CPB use, and those with different dosing regimens. The included patients were administered 1000 mg of TXA at the start of the operative procedure and after CPB was discontinued. The S5 system (Sorin, Mirandola, Italy) was used for the CPB. Target pump flows were set to be 2.0–3.0 L/min/m^2^ with a target mean arterial pressure. Blood samples were obtained at approximately 0.5, 1, 2, and 5 h after TXA administration and at 1, 6, and 16 h after TXA re-administration in heparinized tubes. The samples were centrifuged at 3000 rpm for 5 min to separate the plasma fractions. All samples were stored at − 80 °C until assayed. To remove phospholipids and proteins from the plasma, 300 µL of 1% formic acid in acetonitrile was added to 100 µL of plasma. The supernatants of plasma samples were extracted using a solid-phase extraction cartridge (GL Sciences Inc., Tokyo, Japan). The fraction was diluted in a 100-fold volume (1 mL) with 0.1% formic acid in acetonitrile, and then 100 µL sample was added 20 µL of internal standard (IS).

### Chemicals

TXA [*trans*−4-(aminomethyl)cyclohexanecarboxylic acid] injection and the IS (*cis*−4-aminocyclohexanecarboxylic acid) were obtained from Nichi-Iko Pharmaceutical Co., Ltd. (Toyama, Japan) and Tokyo Chemical Industry Co., Ltd. (Tokyo, Japan), respectively. High-performance liquid chromatography grade formic acid, acetonitrile, and methanol were purchased from FUJIFILM Wako Pure Chemical Co. Ltd. (Osaka, Japan).

### Liquid chromatography-tandem mass spectrometry analysis

Concentrations of TXA in plasma were measured using ultra-performance liquid chromatography (UPLC)-tandem mass spectrometry (Waters ACQUITY UPLC® system, ACQUITY TQD) with reference to several reports [17, 18]. Chromatographic separation of TXA and the IS was conducted using an ACQUITY UPLC® BEH HILIC column (1.7 µm, 2.1 × 100 mm) maintained at 40 °C. The separations utilized gradient elution with eluents A and B, consisting of 0.1% formic acid in ultrapure water and methanol, respectively. The gradient program was as follows: starting with 99.9% eluent A (linear gradient, v/v) at 0 min, 85% eluent A at 2 min, 30% eluent A at 4 min, and returning to 99.9% eluent A at 6 min at a flow rate of 0.2 mL/min. Each injection volume of 20 µL had a runtime of 6 min. Quantitation was performed using multiple reaction monitoring with electrospray ionization in positive mode. The multiple reaction monitoring transitions were as follows: for TXA, from m/z 157.9 to 122.8, 99.7, 68.8, 50.8, and 40.1, and for IS, from m/z 143.9 to 108.8, 81.0, 80.7, 40.8, and 40.4.

### Data collection and assessment

Data were retrospectively collected from the patients’ electronic medical record system (HOPE/EGMAIN-GX; Fujitsu, Tokyo, Japan) and anesthesia recording system databases (ORSYS and ACSYS; Philips Healthcare, Best, Netherlands). Demographic data, including sex, age, body weight (BW), height, and body mass, were collected. Serum creatinine (Scr) levels were measured before the cardiac surgery. Intraoperative data, including the CPB duration, pump prime volume, blood transfusion volume, cell saver volume, and ultrafiltrate volume; type of surgery; and bleeding volume, in the operating room were recorded. Body surface area (BSA) and creatine clearance (CL_cr_) were calculated using the formulas of DuBois and DuBois [19] and the formula of Cockcroft-Gault [20], respectively.

DuBois and DuBois:

$$\begin{array}{c}\text{BSA }({\text{m}}^{2})\\ = (\text{BW }{\left(\text{kg}\right)}^{0.425} \times \text{ height }{\left(\text{cm}\right)}^{0.725}) \times 0.007184,\end{array}$$ 

Cockcroft-Gault:

$$\begin{array}{c}{\text{CL}}_{\text{cr}} (\text{mL}/\text{min}) = \{(140 -\text{ age }(\text{year})) \times \text{ BW }(\text{kg})\} \\ \ \ \ \ \ \ \ \ / (\text{Scr }(\text{mg}/\text{dL}) \times 72 (\text{mg}/\text{dL}))\ (\times 0.85\text{ if female})\end{array}$$ 

### Ethical considerations

This study adhered to the principles of the Declaration of Helsinki and Ethical Guidelines for Medical and Health Research Involving Human Subjects. The study protocol was approved by the Ethics Committee of Nagoya University Hospital (approval no. 2021–0250). Written informed consent was obtained from all enrolled patients. All data were assigned unique identification numbers specific to each insured person, and no personally identifiable information was used in the study. Patient data were anonymized by the database provider, and none of the authors had access to the original data containing personal information.

### Population PK modeling

In this study, a population-compartmental PK model was constructed using a nonlinear mixed-effects (NLME) model. All plasma concentration–time data for TXA were analyzed simultaneously using Phoenix NLME software version 8.4.0 (Certara, Princeton, NJ). Population PK parameters and variability were estimated using the first-order conditional estimation with the extended least-squares method (FOCE-ELS). The stability and predictive ability of the final model were validated using visual prediction testing (VPC) and bootstrapping.

#### Base model

Models with one or two compartments were evaluated using first-order elimination from the central compartment. One-compartment models were parameterized by clearance (CL_1_) and volume of distribution of the central compartment (*V*_1_). In the two-compartment models, additional parameters, such as intercompartment clearance (CL_2_) and volume of distribution of the peripheral compartment (*V*_2_), were used. These models were evaluated using a likelihood ratio test with a change of − 2 log-likelihood difference (− 2 l.l.d.) (the difference in the objective function value [ΔOFV]). If the decrease of ΔOFV obtained from a selected model was more than 6.635 (*χ*^2^; degrees of freedom, 1; *p* < 0.01), the model was considered statistically significant.

Interindividual variability (*η*) of the parameters in the PK model was assumed to be log-normally distributed using an exponential random effect model as follows:

$${P}_{i} = TV(P) \times exp({\eta }_{i})$$

where *P*_i_ is the value of the PK parameter for an individual (*i*th) patient, TV*(P)* is the population mean value of the typical PK parameters, and *η*_i_ represents the deviation of the *P*_i_ from TV*(P)*. The *η*_i_ was assumed to follow a normal distribution with a mean of 0 and a variance of *ω*^2^.

Residual variability (*ε*) was assumed to be log-normally distributed using an additive and multiplicative (combined) error model as follows,

$$Cob{s}_{i,j} = Cpre{d}_{i,j} + {\varepsilon }_{i,j}\times \sqrt{{1+\left({Cpred}_{\text{i},\text{j}} \times {\sigma }_{2}/{\sigma }_{1}\right)}^{2}}$$

where *Cobs*_*i,j*_ and *Cpred*_*i,j*_ represent the observed concentration and predicted concentration for the *i*th patient at time *j*, respectively, *σ*_1_ denotes the standard deviation of *ε*_i,j_, and *σ*_2_ denotes the multiplicative *σ*. The *ε*_i,j_ was assumed to follow a normal distribution with a mean of 0 and a variance of *σ*^2^.

#### Covariate model

Based on several studies [13, 16, 21], BW and CL_cr_ were selected as candidates for PK covariates. After establishing the basic PK model, the effects of the covariates were evaluated using the shotgun method. If a decrease of ΔOFV was greater than 6.635 (*χ*^2^; degrees of freedom, 1; *p* < 0.01) compared to the basic model, the covariate was considered to significantly influence the model parameters. Significant covariates were retained in the model, and non-significant covariates were removed.

### Model evaluation

OFV were used for model diagnostics between the base and final models. Scatter plots were used to investigate the goodness of fit of the models. Model fitting was evaluated by goodness of fit to the observed TXA concentrations versus population predicted concentrations (PRED), observed TXA concentrations versus individually predicted concentrations (IPRED), and conditional weighted residuals (CWRES) versus PRED.

The VPC and bootstrapping were used to validate the final model. To evaluate the predictive performance, VPC was performed using 1000 virtual data sets generated from the final model. VPC confirmed that the observed TXA concentration fell within the 90% prediction interval stimulated by the final population PK model. To evaluate the reliability and stability of the final model, bootstrapping was performed with 1000 datasets generated from repeated sampling of the original datasets. The median, relative standard error (RSE), and 95% confidence interval (2.5–97.5%) for the estimates from the original data set were calculated and compared with the final model estimates.

## Results

### Patient characteristics

In this observational study, a total of 88 patients underwent cardiac surgery and received IV TXA. We excluded eight patients with hemodialysis, two without CPB usage, and one with a different dosing regimen. The remaining 77 patients were included in the PK model. Patient characteristics are presented in Table 1. Patients had a median age of 69 years (range 26–84 years), median weight of 61.4 kg (range 38.0–97.6 kg), and mean BMI of 24.0 kg/m^2^ (range 15.1–35.0 kg/m^2^). Seven patients (9.1%) had a BMI above 30.0 kg/m^2^. With regard to kidney function, patients had a median SCr at 85.7 µmol/L (range 38.0–271.4 µmol/L) and a median CL_Cr_ at 61.0 mL/min (range 21. 8–147.5 mL/min). During surgery, patients had a median CPB duration of 170 min (range 76–424 min), median pump prime volume of 1109 mL (range 809–1409 mL), median cell saver volume of 468 mL (range 11–6409 mL), median ultrafiltrate volume of 3000 mL (range 250–14,200 mL), and median bleeding volume of 496 mL (range 8–7721 mL).

**Table 1:** Characteristics of the patients receiving tranexamic acid

Characteristic	
Male/female (*n*)	51/26
Age (yrs)	69 (60–75)
Body weight (kg)	61.4 (54.6–75.2)
Height (cm)	161.8 (156.5–170.7)
Body surface area (m^2^)	1.7 (0.2)
Body mass index (kg/m^2^)	24.0 (4.2)
Serum creatinine (µmol/L)	85.7 (72.5–105.2)
Creatine clearance^#^ (mL/min)	61.0 (48.1–76.3)
Type of surgery (*n*)	
CABG	16
Single valve^##^	35
Complex valve	2
Aorta*	15
Complex**	6
Other	3
Intraoperative data	
CPB duration (min)	170 (136–210)
Pump prime volume (mL)	1109 (1109–1209)
Blood transfusion (mL)	560 (280–840)
Cell saver (mL)	468 (211–840)
Ultrafiltrate (mL)	3000 (2000–4000)
Bleeding volume (mL)	496.0 (214.0–1000)

Values were presented as numbers, means (standard deviations, SD), or medians (interquartile ranges, IQR). Normally distributed data (as evaluated using the Shapiro–Wilk test) are presented as mean (SD), whereas not normally distributed data are presented as median (IQR)

*AVR* aortic valve replacement, *MVR* mitral valve replacement, *MVP* mitral valve plasty, *CABG* coronary artery bypass grafting, *CPB* cardiopulmonary bypass

*Aortic surgery such as total arch or descending aortic replacement

**Combined surgery, such as MVP + CABG and MVP + MAZE

^#^Creatine clearance was calculated using the Cockcroft-Gault equation with actual body weight ^##^AVR, MVR, MVP, and tricuspid valve replacement

### Population pharmacokinetic analysis

**Fig. 1:**
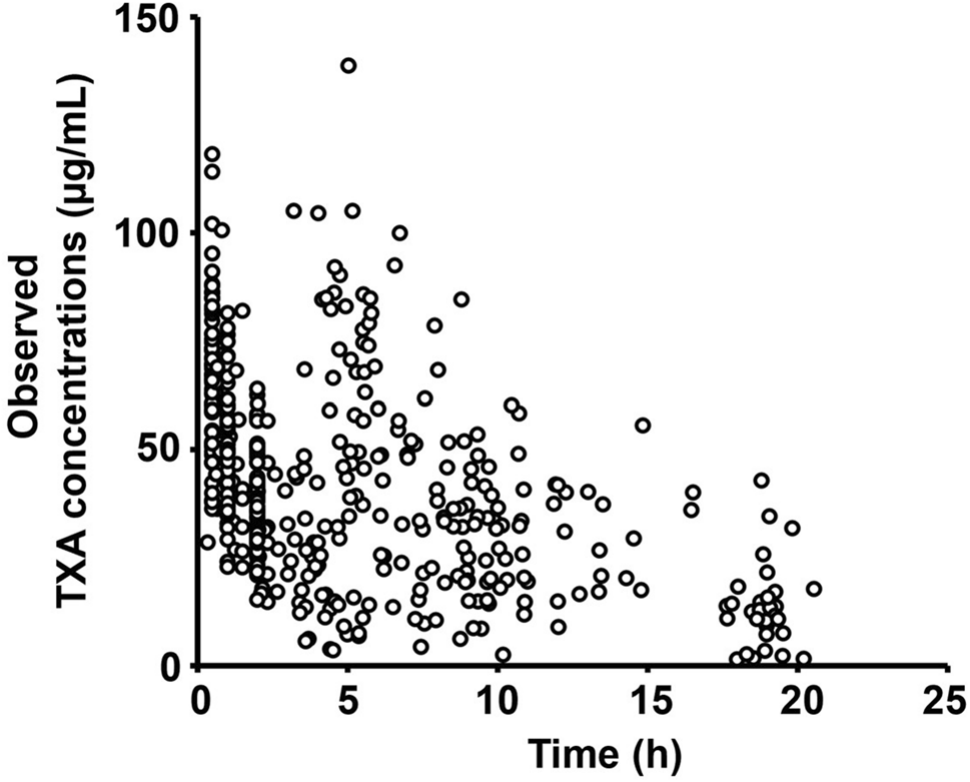
Observed tranexamic acid (TXA) concentration

The TXA concentration data were collected from 77 patients (Fig. 1). TXA concentrations during surgery with CPB ranged from 3.488 to 118.3 µg/mL. The plasma TXA concentrations in all patients after CPB interruption and TXA re-administration ranged from 1.463 to 138.8 µg/mL. Notably, three enrolled patients had < 5 µg/mL plasma TXA concentrations collected during surgery with CPB. The characteristics of the three patients are shown in Supplemental Table S1. A total of 453 blood samples were collected for plasma concentration analysis. In the base model, additive, multiplicative, and combined error models were evaluated, and combined error models were used. The two-compartment-based model with the combined error model was determined to be the best model compared to the one-compartment-based model with the combined error model (Table 2, ΔOFV =  − 109.550). Furthermore, shotgun simulation of the covariates was performed using a two-compartment base model with a combined error model (Table 2). In the final population PK model, BW and CL_cr_ significantly influenced the *V*_1_ and CL_1_ of TXA, respectively. The population estimates in the final model showed that *V*_1_, *V*_2_, CL_1,_ and CL_2_ were 12.77 L, 6.857 L, 3.263 L/h, and 2.859 L/h, respectively (Table 3). In this model, the RSE of all the parameters was 3.582–16.55%. The parameters and relative standard errors of the final PK model and bootstrap results are shown in Table 3. Bootstrap stimulation with 1000 replicates was performed to validate the model. The final model may be summarized as *V*_1_ (L) = 12.77 × (BW/61.4)^0.911^, *V*_2_ (L) = 6.857, CL_1_ (L/h) = 3.263 × [CL_cr_ (L/h) / 61.0]^0.752^, CL_2_ (L/h) = 2.859.

**Table 2:** Results of the population pharmacokinetic model for tranexamic acid

Model no	Model description	OFV	ΔOFV	*p* value
1	Basic model (1-compartment model)	3492.0236		
2	Basic model (2-compartment model)	3382.4731	− 109.550	< 0.01
3	Add BW on CL_2_ in model 2	3369.6756	− 12.7975	< 0.01
4	Add BW on *V*_1_ in model 2	3358.3354	− 24.1377	< 0.01
5	Add CLcr on CL_1_ in model 2	3343.5329	− 38.9402	< 0.01
6	Add CLcr on CL_1_ in model 3	3327.2809	− 42.3948	< 0.01
7	Add CLcr on CL_1_ in model 4	3312.9365	− 45.3989	< 0.01

*OFV* objective function value, *BW* body weight, *CL* clearance, *V* volume of distribution, *CLcr* creatinine clearance

**Table 3:** Tranexamic acid population pharmacokinetic variables in 77 patients

Parameters	Final model		Final bootstrap (*N* = 1000)			
	%RSE	Estimate	%RSE	Median	Lower 2.5%	Upper 97.5%
*V*_1_ (L)	3.582	12.77	9.452	13.07	11.24	15.74
*V*_2_ (L)	13.82	6.857	107.6	8.198	5.581	101.0
CL_1_ (L/h)	4.198	3.263	29.69	3.058	1.229	3.608
CL_2_ (L/h)	13.93	2.859	23.29	2.701	1.920	4.171
BW on *V*_1_ (L)	16.51	0.911	20.03	0.893	0.573	1.266
CLcr on CL_1_ (L/h)	16.55	0.752	40.75	0.920	0.591	2.052

*%RSE* percentage relative standard error, *V* volume of distribution, *CL* clearance, *BW* body weight, *CLcr* creatinine clearance

**Fig. 2:**
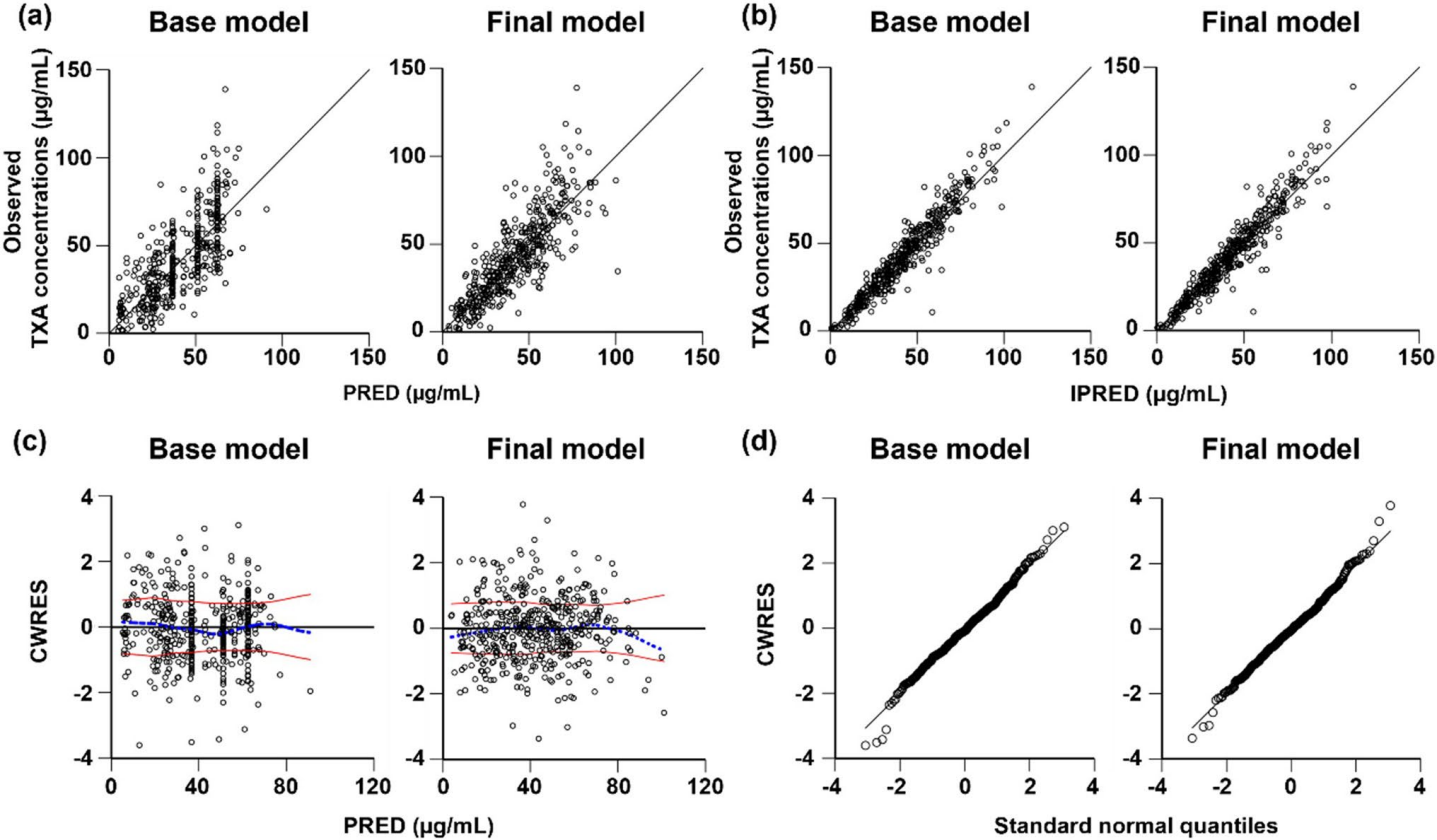
Diagnostic goodness-of-fit plots of base model and final model. **a** The observed TXA plasma concentrations versus PRED; **b** the observed TXA plasma concentrations versus IPRED; **c** The CWRES versus PRED, the blue dot line is the overall trend of data fitting, and two red solid lines are the absolute value distribution of the data; **d** the CWRES versus standard normal quantiles. TXA, tranexamic acid; PRED, population predicted concentrations; IPRED, individually predicted concentrations; CWRES, conditional weighted residuals

The goodness-of-fit diagnostic plots for the base and final models are shown in Fig. 2. The scatter plot of the observed TXA concentrations versus PRED showed that the plots in the final model were distributed closer along the line of identity compared to the basic model (Fig. 2a), resulting in population TXA concentrations being well predicted in the final model. In addition, the scatter plot of observed TXA concentrations versus IPRED in the base model was similar to that of the final model (Fig. 2b). In both the base model and final model, the plots of CWRES versus PRED were distributed within ± 2 standard deviations of the mean, indicating that there was no clear bias between PRED and CWRES (Fig. 2c). The *η* and *ε* of the basal model and final model were evaluated using the standard normal quantile plots of CWRES (Fig. 2d). The plots resemble a normal distribution, which agrees with the modeling assumptions.

Bootstrap results of the final PK model validation are listed in Table 3. For all distribution parameters, the bootstrap medians were similar to the final model estimate. The bootstrap parameter variables and final model were all within 95% confidence intervals, although the %RSE of *V*_2_ was high. This result suggests that the final model was stable.

## Discussion

TXA is an effective antifibrinolytic agent in cardiac surgery that reduces the risk of blood loss in patients [4]. Although various studies have reported the PK properties of TXA, there are few reports on the population PK of TXA in patients undergoing cardiac surgery with CPB. Therefore, we investigated the PK of TXA in patients who underwent cardiac surgery with CPB. We found that the PK of TXA was altered by increasing *V*_1_ and decreasing CL_1_ in patients with CPB compared with that previously reported in patients not treated with CPB. We further identified BW and CL_cr_ as the two covariate determinants affecting TXA concentrations in these patients. Accordingly, TXA dosing with CPB should be considered based on the BW and CL_cr_ in patients who receive TXA.

In the present study, the PK model of TXA in patients who underwent cardiac surgery with CPB fitted well to the two-compartment–based model with the combined error model (Table 2). These results were supported by the fact that the median bootstrap values for all distribution parameters were mostly similar to the final model estimates (Table 3). Various studies have also reported that the PK model of TXA is best described by a two-compartment–based model [13, 21–26]. These findings are consistent with our results. Assuming a body weight of 61.4 kg (median weight) and CL_cr_ of 60.0 mL/min, our model estimates the CL_1_ as 3.223 L/h (53.72 mL/min) and as *V*_1_ 12.77 L. These data show that CL_1_ was noticeably lower and *V*_1_ was higher than those in the product information data (CL_1_, 110–116 mL/min; *V*_1_, 9–12 L). This trend of PK alterations is often reported in CPB patients receiving hydrophilic drugs with a smaller volume of distribution such as TXA and cephalosporins, which are mainly removed via renal excretion [16, 27, 28]. The PK parameters of drugs are altered by several aspects of CPB, including drug sequestration by the CPB circuit, increased volume of distribution, changes in protein binding, tissue distribution, and hemodilution [27, 28]. Dowd et al. reported that CPB induced a decrease in CL_1_ and an increase in *V*_1_ for a 70-kg patient who underwent cardiac surgery, suggesting that CPB affected TXA PK [16]. The present results also suggest that dose adjustment tailored to CPB is required to achieve therapeutic TXA levels in patients undergoing cardiac surgery with CPB.

Previous studies have reported that the TXA population estimates were 4.8–6.6 L/h for CL_1_, 5.0–11.9 L for *V*_1_, 12.6–32.2 L/h for CL_2_, and 9.1–10.8 L for *V*_2_ in 70- to 85-kg adult patients who undergo cardiac surgery with CPB [13, 16, 24]. In the present population PK model, the estimates of CL_1_, *V*_1_, CL_2,_ and *V*_2_ were 3.263 L/h, 12.77 L, 2.859 L/h, and 6.857 L, respectively, at a BW of 61.4 kg (median) and CL_cr_ 61.0 mL/min (median) in a patient who underwent cardiac surgery with CPB. Accordingly, the present results suggest no marked differences in CL_1_ and *V*_1_ between our data and theirs. However, the estimates of CL_2_ and *V*_2_ in the present study were lower than those previously reported [13, 16, 24]. This result may be attributed to the different CPB devices, types of surgery, and TXA dosing regimens used in the enrolled patients (Table 1). In addition, their TXA PK model cannot be applied to patients with renal failure who undergo cardiac surgery with CPB because the model included only BW as a covariate. The model presented in the current study was adjusted for both BW and CL_cr_. Therefore, our model can be more flexibly adapted for these patients.

Andersson et al. reported that fibrinolysis (lysis area on plate) was reduced by 98% with 100 mg/L TXA, 90% with 25 mg/L TXA, and 80% with 10 mg/L TXA in fibrin plate analysis with tissue homogenates containing tPA [29]. Dowd et al. hypothesized that TXA must be administered so that the plasma concentration of TXA is exceeded at all times at a plateau concentration of 126 µg/mL to achieve a stable effect during and in the immediate postoperative period [13, 16]. However, the risk of seizures is higher with a total perioperative dose of 100 mg/kg than with a total dose of 50 mg/kg [4, 30]. A meta-study showed that the risk of seizures increases with increasing doses and is higher in patients receiving > 2 g of TXA per day [31]. Therefore, considering the risk of seizures at higher doses with reference to these reports, physicians may prefer to administer the lowest dose necessary to maintain effective TXA levels in circulation.

Strauss et al. hypothesized that a double-bolus regimen with a 1 g bolus of TXA in the CPB priming solution and another 1 g bolus before protamine administration would maintain a target plasma level at or above 15 mg/L once CPB was started and provide peak antifibrinolytic effect post-CPB [24]. Their report indicated that TXA concentrations in 20 patients were in the range of 28.3–255.0 mg/L during CPB and 17.6–175.4 mg/L from 5 min post-CPB to 2 h after infusion. Similar to their regimen, a double-bolus regimen with a 1 g bolus of TXA at the start of the operative procedure and after CPB was discontinued for patients undergoing cardiac surgery with CPB at our institution, although the approved maximum TXA dose in Japan is 2.5 g/day. We demonstrated that TXA concentrations in 77 patients were in the range 3.488–118.3 µg/mL during surgery with CPB and 1.463–138.8 µg/mL after CPB interruption and TXA re-administration (Fig. 1). These results showed that the TXA concentrations of the patients reported in the present study were lower overall than those reported previously. This may also be attributed to the different types of surgery, CPB devices, CPB duration, and CPB management in the enrolled patients (Table 1).

Plasma TXA concentrations collected during surgery with CPB in most enrolled patients exceeded 5 µg/mL in this study. Many studies have stated that the effective TXA plasma concentration should be stable and greater than 10–15 µg/mL [15, 32–37], although concentrations between 5 and 10 µg/mL partly inhibit fibrinolysis [37–39]. Another study reported that TXA in doses of < 2 g and 2–10 g reduced transfusion rates similarly [1]. Fiechtner et al. demonstrated that 21 cardiac surgery patients undergoing CPB who received an initial TXA dose of 10 mg/kg IV over 20 min and then 1 mg/kg/h through a central line until 20 h post-ICU admission had effective serum TXA levels above 10 µg/mL at all measured time points [40]. These results suggest that the TXA dose regimen at our institution has some positive effects on maintaining a minimum effective TXA concentration and reducing the risk of seizures for patients undergoing cardiac surgery with CPB.

In this study, three enrolled patients had < 5 µg/mL plasma TXA concentrations during surgery with CPB (Supplemental Table S1). Of the three patients, one (patient ID: 28) had a longer CPB duration at 424 min than the other enrolled patients. Another patient (patient ID: 7) had a higher bleeding volume of 1627 mL. For the other patient (patient ID: 5), we were unable to identify the cause of the lower plasma TXA concentration. For at least two patients, the long CPB time and high bleeding volume were probably responsible for the low plasma TXA concentrations.

The present study has a few limitations. This was a single-center study with a small sample size (*N* = 77). Collecting all plasma samples from the patients was difficult because some patients were administered TXA prior to blood collection. For example, most patients undergoing CABG and valve surgeries were separated from CPB within 5 h and TXA was re-administered before 5 h. Additionally, sampling times were sparse, which may have affected the accuracy of the PK estimates. However, judging from the validation assessment of the final model, the present model appears to provide good estimates of the PK parameters predicting TXA concentrations. Another limitation is that a comparison between CPB and non-CPB patients was not conducted owing to the lack of a control group.

## Conclusions

We performed TXA population PK analysis to determine the plasma TXA concentration in patients who underwent cardiac surgery with CPB. Furthermore, a population TXA PK model was successfully established for these patients. The final model included the effect of BW and CL_cr_ may be summarized as follows: *V*_1_ (L) = 12.77 × (BW/61.4)^0.911^, *V*_2_ (L) = 6.857, CL_1_ (L/h) = 3.263 × [CL_cr_ (L/h) / 61.0]^0.752^, CL_2_ (L/h) = 2.859. Dose adjustment of TXA tailored to CPB may be required to achieve sufficient treatment effects in patients who undergo cardiac surgery with CPB. This model may be useful for the development of individualized TXA dosing regimens in patients undergoing cardiac surgery with CPB. Future studies in larger populations with non-CPB groups are required to validate the results of the present study.

## Supplementary Information

Below is the link to the electronic supplementary material.

Supplementary file1 (DOCX 300 KB)

## Data Availability

Data are provided within the manuscript. The data that support the findings of this study are available from the corresponding authors upon reasonable request.

## References

[1] Henry DA, Carless PA, Moxey AJ, O’Connell D, Stokes BJ, Fergusson DA, Ker K (2011) Anti-fibrinolytic use for minimising perioperative allogeneic blood transfusion. Cochrane Database Syst Rev 3:CD001886. 10.1002/14651858.CD001886.pub4 10.1002/14651858.CD001886.pub4 PMC4234031 21412876

[2] Ker K, Edwards P, Perel P, Shakur H, Roberts I (2012) Effect of tranexamic acid on surgical bleeding: systematic review and cumulative meta-analysis. BMJ 344:e3054. 10.1136/bmj.e3054 22611164 10.1136/bmj.e3054 PMC3356857

[3] Mannucci PM (1998) Hemostatic drugs. N Engl J Med 339(4):245–253. 10.1056/NEJM199807233390407 9673304 10.1056/NEJM199807233390407

[4] Myles PS, Smith JA, Forbes A, Silbert B, Jayarajah M, Painter T, Cooper DJ, Marasco S, McNeil J, Bussières JS, McGuinness S, Byrne K, Chan MTV, Landoni G, Wallace S, ATACAS Investigators of the ANZCA Clinical Trials Network (2017) Tranexamic acid in patients undergoing coronary-artery surgery. N Engl J Med 376(2):136–148. 10.1056/NEJMoa1606424 27774838 10.1056/NEJMoa1606424

[5] Bartlett RH (2005) Extracorporeal life support: history and new directions. ASAIO J 51(5):487–489. 10.1097/01.mat.0000179141.08834.cb 16322701 10.1097/01.mat.0000179141.08834.cb

[6] Bartlett RH, Gattinoni L (2010) Current status of extracorporeal life support (ECMO) for cardiopulmonary failure. Minerva Anestesiol 76(7):534–540 20613694

[7] Karkouti K, McCluskey SA, Syed S, Pazaratz C, Poonawala H, Crowther MA (2010) The influence of perioperative coagulation status on postoperative blood loss in complex cardiac surgery: a prospective observational study. Anesth Analg 110(6):1533–1540. 10.1213/ANE.0b013e3181db7991 20435945 10.1213/ANE.0b013e3181db7991

[8] Ngaage DL, Bland JM (2010) Lessons from aprotinin: is the routine use and inconsistent dosing of tranexamic acid prudent? Meta-analysis of randomised and large matched observational studies. Eur J Cardiothorac Surg 37(6):1375–1383. 10.1016/j.ejcts.2009.11.055 20117944 10.1016/j.ejcts.2009.11.055

[9] Tsementzis SA, Meyer CH, Hitchcock ER (1992) Cerebral blood flow in patients with a subarachnoid haemorrhage during treatment with tranexamic acid. Neurochirurgia (Stuttg) 35(3):74–78. 10.1055/s-2008-1052251 1603224 10.1055/s-2008-1052251

[10] Keyl C, Uhl R, Beyersdorf F, Stampf S, Lehane C, Wiesenack C, Trenk D (2011) High-dose tranexamic acid is related to increased risk of generalized seizures after aortic valve replacement. Eur J Cardiothorac Surg 39(5):e114–e121. 10.1016/j.ejcts.2010.12.030 21295991 10.1016/j.ejcts.2010.12.030

[11] Manji RA, Grocott HP, Leake J, Ariano RE, Manji JS, Menkis AH, Jacobsohn E (2012) Seizures following cardiac surgery: the impact of tranexamic acid and other risk factors. Can J Anaesth 59(1):6–13. 10.1007/s12630-011-9618-z 22065333 10.1007/s12630-011-9618-z

[12] Murkin JM, Falter F, Granton J, Young B, Burt C, Chu M (2010) High-dose tranexamic acid is associated with nonischemic clinical seizures in cardiac surgical patients. Anesth Analg 110(2):350–353. 10.1213/ANE.0b013e3181c92b23 19996135 10.1213/ANE.0b013e3181c92b23

[13] Grassin-Delyle S, Tremey B, Abe E, Fischler M, Alvarez JC, Devillier P, Urien S (2013) Population pharmacokinetics of tranexamic acid in adults undergoing cardiac surgery with cardiopulmonary bypass. Br J Anaesth 111(6):916–924. 10.1093/bja/aet255 23880099 10.1093/bja/aet255

[14] Karski JM, Dowd NP, Joiner R, Carroll J, Peniston C, Bailey K, Glynn MF, Teasdale SJ, Cheng DC (1998) The effect of three different doses of tranexamic acid on blood loss after cardiac surgery with mild systemic hypothermia (32 degrees C). J Cardiothorac Vasc Anesth 12(6):642–646. 10.1016/s1053-0770(98)90235-x 9854660 10.1016/s1053-0770(98)90235-x

[15] Sigaut S, Tremey B, Ouattara A, Couturier R, Taberlet C, Grassin-Delyle S, Dreyfus JF, Schlumberger S, Fischler M (2014) Comparison of two doses of tranexamic acid in adults undergoing cardiac surgery with cardiopulmonary bypass. Anesthesiology 120(3):590–600. 10.1097/ALN.0b013e3182a443e8 23903022 10.1097/ALN.0b013e3182a443e8

[16] Dowd NP, Karski JM, Cheng DC, Carroll JA, Lin Y, James RL, Butterworth J (2002) Pharmacokinetics of tranexamic acid during cardiopulmonary bypass. Anesthesiology 97(2):390–399. 10.1097/00000542-200208000-00016 12151929 10.1097/00000542-200208000-00016

[17] Fabresse N, Fall F, Etting I, Devillier P, Alvarez JC, Grassin-Delyle S (2017) LC-MS/MS determination of tranexamic acid in human plasma after phospholipid clean-up. J Pharm Biomed Anal 141:149–156. 10.1016/j.jpba.2017.04.024 28445815 10.1016/j.jpba.2017.04.024

[18] Ivica J, Gauthier J, Power P, Lamy A, Potter M (2021) Analysis of serum tranexamic acid in patients undergoing open heart surgery. Clin Biochem 87:74–78. 10.1016/j.clinbiochem.2020.10.010 33188769 10.1016/j.clinbiochem.2020.10.010

[19] DuBois D, DuBois EF (1916) A formula to estimate theapproximate surface area if height and weight be known. Arch Intern Med 17:863–871. 10.1001/archinte.1916.00080130010002

[20] Cockcroft DW, Gault MH (1976) Prediction of creatinine clearance from serum creatinine. Nephron 16(1):31–41. 10.1159/000180580 1244564 10.1159/000180580

[21] Goobie SM, Meier PM, Sethna NF, Soriano SG, Zurakowski D, Samant S, Pereira LM (2013) Population pharmacokinetics of tranexamic acid in paediatric patients undergoing craniosynostosis surgery. Clin Pharmacokinet 52(4):267–276. 10.1007/s40262-013-0033-1 23371895 10.1007/s40262-013-0033-1

[22] Grassin-Delyle S, Semeraro M, Lamy E, Urien S, Runge I, Foissac F, Bouazza N, Treluyer JM, Arribas M, Roberts I, Shakur-Still H (2022) Pharmacokinetics of tranexamic acid after intravenous, intramuscular, and oral routes: a prospective, randomised, crossover trial in healthy volunteers. Br J Anaesth 128(3):465–472. 10.1016/j.bja.2021.10.054 34998508 10.1016/j.bja.2021.10.054

[23] Li S, Ahmadzia HK, Guo D, Dahmane E, Miszta A, Luban NLC, Berger JS, James AH, Wolberg AS, van den Anker JN, Gobburu JVS (2021) Population pharmacokinetics and pharmacodynamics of tranexamic acid in women undergoing caesarean delivery. Br J Clin Pharmacol 87(9):3531–3541. 10.1111/bcp.14767 33576009 10.1111/bcp.14767 PMC8355246

[24] Strauss ER, Li S, Henderson R, Carpenter R, Guo D, Thangaraju K, Katneni U, Buehler PW, Gobburu JVS, Tanaka KA (2022) A pharmacokinetic and plasmin-generation pharmacodynamic assessment of a tranexamic acid regimen designed for cardiac surgery with cardiopulmonary bypass. J Cardiothorac Vasc Anesth 36(8 Pt A):2473–2482. 10.1053/j.jvca.2021.12.029 35094925 10.1053/j.jvca.2021.12.029

[25] Stitt G, Spinella PC, Bochicchio GV, Roberts I, Downes KJ, Zuppa AF (2024) Population pharmacokinetic modelling and simulation of tranexamic acid in adult trauma patients. Br J Clin Pharmacol 90:1932–1941. 10.1111/bcp.16075 38697615 10.1111/bcp.16075 PMC11932107

[26] Wesley MC, Pereira LM, Scharp LA, Emani SM, McGowan FX Jr, DiNardo JA (2015) Pharmacokinetics of tranexamic acid in neonates, infants, and children undergoing cardiac surgery with cardiopulmonary bypass. Anesthesiology 122(4):746–758. 10.1097/ALN.0000000000000570 25585004 10.1097/ALN.0000000000000570

[27] Mets B (2000) The pharmacokinetics of anesthetic drugs and adjuvants during cardiopulmonary bypass. Acta Anaesthesiol Scand 44(3):261–273. 10.1034/j.1399-6576.2000.440308.x 10714838 10.1034/j.1399-6576.2000.440308.x

[28] Rosen DA, Rosen KR (1997) Elimination of drugs and toxins during cardiopulmonary bypass. J Cardiothorac Vasc Anesth 11(3):337–340. 10.1016/s1053-0770(97)90104-x 9161903 10.1016/s1053-0770(97)90104-x

[29] Andersson L, Nilsoon IM, Colleen S, Granstrand B, Melander B (1968) Role of urokinase and tissue activator in sustaining bleeding and the management thereof with EACA and AMCA. Ann N Y Acad Sci 146(2):642–658. 10.1111/j.1749-6632.1968.tb20322.x 5254275 10.1111/j.1749-6632.1968.tb20322.x

[30] Couture P, Lebon JS, Laliberté É, Desjardins G, Chamberland M, Ayoub C, Rochon A, Cogan J, Denault A, Deschamps A (2017) Low-dose versus high-dose tranexamic acid reduces the risk of nonischemic seizures after cardiac surgery with cardiopulmonary bypass. J Cardiothorac Vasc Anesth 31(5):1611–1617. 10.1053/j.jvca.2017.04.026 28803773 10.1053/j.jvca.2017.04.026

[31] Murao S, Nakata H, Roberts I, Yamakawa K (2021) Effect of tranexamic acid on thrombotic events and seizures in bleeding patients: a systematic review and meta-analysis. Crit Care 25(1):380. 10.1186/s13054-021-03799-9 34724964 10.1186/s13054-021-03799-9 PMC8561958

[32] Eriksson O, Kjellman H, Pilbrant A, Schannong M (1974) Pharmacokinetics of tranexamic acid after intravenous administration to normal volunteers. Eur J Clin Pharmacol 7(5):375–380. 10.1007/bf00558210 4422030 10.1007/BF00558210

[33] Horrow JC, Van Riper DF, Strong MD, Grunewald KE, Parmet JL (1995) The dose-response relationship of tranexamic acid. Anesthesiology 82(2):383–392. 10.1097/00000542-199502000-00009 7856897 10.1097/00000542-199502000-00009

[34] Nuttall GA, Gutierrez MC, Dewey JD, Johnson ME, Oyen LJ, Hanson AC, Oliver WC Jr (2008) A preliminary study of a new tranexamic acid dosing schedule for cardiac surgery. J Cardiothorac Vasc Anesth 22(2):230–235. 10.1053/j.jvca.2007.12.016 18375325 10.1053/j.jvca.2007.12.016

[35] Pilbrant A, Schannong M, Vessman J (1981) Pharmacokinetics and bioavailability of tranexamic acid. Eur J Clin Pharmacol 20(1):65–72. 10.1007/bf00554669 7308275 10.1007/BF00554669

[36] Soslau G, Horrow J, Brodsky I (1991) Effect of tranexamic acid on platelet ADP during extracorporeal circulation. Am J Hematol 38(2):113–119. 10.1002/ajh.2830380208 1951300 10.1002/ajh.2830380208

[37] Picetti R, Shakur-Still H, Medcalf RL, Standing JF, Roberts I (2019) What concentration of tranexamic acid is needed to inhibit fibrinolysis? A systematic review of pharmacodynamics studies. Blood Coagul Fibrinolysis 30(1):1–10. 10.1097/MBC.0000000000000789 30585835 10.1097/MBC.0000000000000789 PMC6365258

[38] He S, Johnsson H, Zabczyk M, Hultenby K, Cao H, Blombäck M (2013) A fibrinogen concentrate Haemocomplettan (Riastap) or a factor XIII concentrate Fibrogammin combined with a mini dose of tranexamic acid can reverse the fibrin instability to fibrinolysis induced by thrombin- or FXa-inhibitor. Br J Haematol 160(6):806–816. 10.1111/bjh.12189 23360261 10.1111/bjh.12189

[39] Niego B, Horvath A, Coughlin PB, Pugsley MK, Medcalf RL (2008) Desmoteplase-mediated plasminogen activation and clot lysis are inhibited by the lysine analogue tranexamic acid. Blood Coagul Fibrinolysis 19(4):322–324. 10.1097/MBC.0b013e3282f54568 18469556 10.1097/MBC.0b013e3282f54568

[40] Fiechtner BK, Nuttall GA, Johnson ME, Dong Y, Sujirattanawimol N, Oliver WC Jr, Sarpal RS, Oyen LJ, Ereth MH (2001) Plasma tranexamic acid concentrations during cardiopulmonary bypass. Anesth Analg 92(5):1131–11136. 10.1097/00000539-200105000-00010 11323334 10.1097/00000539-200105000-00010

