# Supplementary material for: Population pharmacokinetic model of tranexamic acid in patients who undergo cardiac surgery with cardiopulmonary bypass
Source: Eur J Clin Pharmacol. 2025 Jan 16;81(3):441–9. doi: 10.1007/s00228-025-03802-0 (PMC11825552; doi:10.1007/s00228-025-03802-0)
Supplement: Supplementary file 1 — Supplementary file1 (DOCX 300 KB) [file 228_2025_3802_MOESM1_ESM.docx]

**Population pharmacokinetic model of tranexamic acid in patients who undergo cardiac surgery with cardiopulmonary bypass**

Tsuyoshi Nakai^1,2,*^, Takahiro Tamura^3^, Yasuhiro Miyagawa^2,*^, Takayuki Inagaki^4^, Masato Mutsuga^5^, Shigeki Yamada^1^, Kiyofumi Yamada^2,6^, Kimitoshi Nishiwaki^3^, Hiroyuki Mizoguchi^2^

^1^Department of Pharmacotherapeutics and Informatics, Fujita Health University School of Medicine, 1-98 Dengakugakubo, Kutsukake-cho, Toyoake, Aichi 470-1192, Japan

^2^Department of Neuropsychopharmacology and Hospital Pharmacy, Nagoya University Graduate School of Medicine, 65 Tsurumai-cho, Showa-ku, Nagoya 466-8560, Japan

^3^Department of Anesthesiology, Nagoya University Graduate School of Medicine, 65 Tsurumai-cho, Showa-ku, Nagoya 466-8550, Japan

^4^Division of Pharmaceutical Sciences I, Faculty of Pharmacy, Meijo University, 150 Yagotoyama, Tempaku-ku, Nagoya 468-8503, Japan

^5^Department of Cardiac Surgery, Nagoya University Graduate School of Medicine, 65 Tsurumai-cho, Showa-ku, Nagoya 466-8550, Japan

^6^Division of Behavioral Neuropharmacology, International Center for Brain Science, Fujita Health University, 1-98 Dengakugakubo, Kutsukake-cho, Toyoake, Aichi 470-1192, Japan

^*^Correspondence:

Tsuyoshi Nakai, Ph.D.

ORCID: 0009-0005-2667-7057

Department of Pharmacotherapeutics and Informatics, Fujita Health University School of Medicine, 1-98 Dengakugakubo, Kutsukake-cho, Toyoake, Aichi 470-1192, Japan

Tel: +81-562-93-9563

Fax: +81-562-93-4593

E-mail: [tsuyoshi.nakai@fujita-hu.ac.jp](mailto:tsuyoshi.nakai@fujita-hu.ac.jp)

Yasuhiro Miyagawa, Ph.D.

Department of Neuropsychopharmacology and Hospital Pharmacy, Nagoya University Graduate School of Medicine, 65 Tsurumai-cho, Showa-ku, Nagoya 466-8560, Japan

Tel.: +81-52-744-2674

Fax: +81-52-744-2979

E-mail: [miyagawa.yasuhiro.x0@f.mail.nagoya-u.ac.jp](mailto:miyagawa.yasuhiro.x0@f.mail.nagoya-u.ac.jp)

**Supplementary material**

**Supplemental Table S1**Characteristics of three patients who had < 5 µg/mL plasma tranexamic acid concentrations during surgery with CPB

*ID* identification; *CABG* coronary artery bypass graft; *CPB* cardiopulmonary bypass. #Creatine clearance was calculated using the Cockcroft-Gault equation with actual body weight. *Aortic surgery such as total arch replacement or descending aortic replacement.
